# Characterization of the T‐cell receptor beta chain repertoire in tumor‐infiltrating lymphocytes

**DOI:** 10.1002/cam4.828

**Published:** 2016-07-27

**Authors:** Katsumi Nakanishi, Yoji Kukita, Hidenobu Segawa, Norimitsu Inoue, Masayuki Ohue, Kikuya Kato

**Affiliations:** ^1^ Department of Molecular and Medical GeneticsResearch InstituteOsaka Medical Center for Cancer and Cardiovascular DiseasesOsakaJapan; ^2^ Department of Tumor ImmunologyResearch InstituteOsaka Medical Center for Cancer and Cardiovascular DiseasesOsakaJapan; ^3^ Department of SurgeryOsaka Medical Center for Cancer and Cardiovascular DiseasesOsakaJapan

**Keywords:** Barcode sequences, colorectal cancer, next‐generation sequencing, T‐cell receptor, Tumor‐infiltrating lymphocytes

## Abstract

Tumor‐infiltrating lymphocytes (TILs) are direct effectors of tumor immunity, and their characterization is important for further development of immunotherapy. Recent advances in high‐throughput sequencing technologies have enabled a comprehensive analysis of T‐cell receptor (TCR) complementarity‐determining region 3 (CDR3) sequences, which may provide information of therapeutic importance. We developed a high‐fidelity target sequencing method with the ability for absolute quantitation, and performed large‐scale sequencing of TCR beta chain (TCRB) CDR3 regions in TILs and peripheral blood lymphocytes (PBLs). The estimated TCRB repertoire sizes of PBLs from four healthy individuals and TILs from four colorectal cancer tissue samples were 608,664–1,003,098 and 90,228–223,757, respectively. The usage of J‐ and V‐regions was similar in PBLs and TILs. Proportions of CDR3 amino acid (aa) sequences occupying more than 0.01% of the total molecular population were 0.33–0.43% in PBLs and 1.3–3.6% in TILs. Additional low coverage sequencing of 15 samples identified five CDR3 aa sequences that were shared by nine patients, one sequence shared by 10 patients, and one sequence shared by 12 patients. The estimated size of the TCRB repertoire in TILs was significantly smaller than that in PBLs. The proportion of abundant species (>0.01%) in TILs was larger than that in PBLs. Shared CDR3 aa sequences represent a response to common antigens, and the identification of such CDR3 sequences may be beneficial in developing clinical biomarkers.

## Introduction

Tumor‐infiltrating lymphocytes (TILs) are a group of lymphocytes present in tumor tissues. TILs interact most closely with tumor cells and are likely to more accurately reflect tumor–host interactions. All types of lymphocytes (i.e., natural killer cells, B cells, and various subtypes of T cells including T helper [Th] 1 cells, Th2 cells, Th17 cells, regulatory T [Treg] cells, and cytotoxic T cells) infiltrate into tumor tissues 1. A strong accumulation of TILs, including CD8^+^ T cells and Th1 cells, is often associated with better outcomes in many kinds of tumors 2, 3. In contrast, some populations of TILs, such as Th2 cells and Treg cells, are sometimes correlated with a poor prognosis, leading to contradictory results. For therapeutic purposes, tumor‐reactive T cells generated from TILs have been used for adoptive cell transfer therapy and the identification of T‐cell receptor (TCR) genes and tumor antigens recognized by the T cells 4 to treat malignancies, including melanoma 5. The recent introduction of immune checkpoint inhibitors 6 is changing the clinical practice of cancer treatment. These agents also activate cytotoxic T cells to act on cancer cells.

T lymphocyte actions depend on the recognition of antigens mediated by the interaction of cell surface molecules (i.e., the heterodimeric T‐cell receptor [TCR]) and a protein degradation product presented by the major histocompatibility complex (MHC) 7. To enable the recognition of diverse peptide–MHC complexes, the T‐cell receptor beta chain (TCRB) locus undergoes somatic recombination among the variable (V), diversity (D), and joining (J) gene segments with the addition/subtraction of bases at recombination junctions. In contrast, the TCR alpha chain locus undergoes VJ recombination, resulting in a limited diversity. The intersection of these specific segments corresponds to the complementarity‐determining region 3 (CDR3) that is important for the recognition of peptide–MHC complexes.

Recent advances in high‐throughput sequencing technologies have enabled the identification of TCR types involved in tumor immunity by large‐scale sequencing of CDR3. One possible application is the use of a TCR type or a group of TCR types as a marker to predict clinical parameters such as prognosis. For these studies, the detailed characterization of TCR types in TILs as a population (the TCR repertoire) is essential. Thus, CDR3 sequences that appear in multiple individuals (“public” sequences) are important for possible practical applications.

The main technical hurdle for sequencing CDR3 is the high error rate of the massively parallel DNA sequencers 8. In previous studies, various methods were used to handle sequencing information 9, 10, 11. Recently, we developed a high‐fidelity target sequencing method based on non‐overlapping integrated reads (NOIRs) generated from multiple sequence reads from individual molecules identified using barcode sequences 12. The NOIR sequencing system (NOIR‐SS) is a high‐fidelity sequencing system based on NOIR and would simplify data processing due to the extremely low error rate. NOIR‐SS can also count the number of sequenced molecules, thereby making it possible to employ a quantitative approach to the TCR repertoire. Due to the high complexity of the TCRB, we first applied the system to the TCRB CDR3 and characterized the TCRB repertoire in TILs compared with those in peripheral blood lymphocytes (PBLs). We also characterized the sharing of CDR3 sequences by TILs from multiple patients.

## Materials and Methods

### Samples

Human peripheral blood samples were obtained from four healthy male volunteers. Colorectal cancer tissues were frozen and stored at −80°C after surgery. Written informed consent was obtained from all patients. This study was approved by the ethics committee of Osaka Medical Center for Cancer and Cardiovascular Diseases.

### RNA extraction

Peripheral blood samples (5 mL) were obtained on two separate drawings from each donor. Heparin was used as the anticoagulant agent. Peripheral blood mononuclear cells (PBMC) including T cells were separated from other blood cell types by Ficoll‐Paque PLUS (GE Healthcare, Little Chalfont, Buckinghamshire, U.K.), according to the manufacturer's instructions, except that we used phosphate buffered saline (PBS) instead of the “Balanced Salt Solution” described in the procedure. Total RNA was isolated from the separated PBMC with TRIzol reagent (Life Technologies, Waltham, MA), according to the manufacturer's instructions. Total RNA from colorectal cancer tissues was extracted from 10 serial 20 *μ*m slices of frozen tissue with TRIzol reagent. The RNA extraction of colorectal cancer tissues was performed with five different sets of serial sections. All extracted RNAs were processed by DNase I and then repurified with the TRIzol reagent. The RNA quality was verified with the Agilent 2100 Bioanalyzer System (Agilent Technologies, Santa Clara, CA). The RNA integrity numbers (RIN) of all samples (an indicator to confirm intact RNA) were >7.0 except for CC06 and CC23 (RIN ≥6.5).

### Primers and adaptors

We designed specific primers for the CDR3 of TCRB. A spacer region consisting of 12 random bases to distinguish molecules, five bases to identify samples (individual index), and an A‐adaptor sequence for ion torrent sequencing was added to the 5′‐end of the TCRB C‐region primer designed by Warren et al. 13. The sequence of the primer TCRB‐CA was 5′‐CCATCTCATCCCTGCGTGTCTCCGACTCAG—XXXXX—NNNNNNNNNNNN—GTACATATTGTCGTT—CTCTGCTTCTGATGGCTCAAAC‐3′ (A‐adaptor—individual‐index designed for the Ion Torrent Sequencers—random barcode—spacer—TCRB C‐region primer). For the V‐region primers, we used the primer sequences of Robins et al. 10. The V‐region primer set with the P1‐adaptor sequence for Ion Torrent sequencing (V‐P1 primer set) is shown in Table S1.

### Sequencing library preparation

For reverse transcription of RNA from blood, 78 *μ*L of solution containing 300 ng of total RNA from PBMCs, 60 pmol of the TCRB‐CA primer, and 0.77 mmol/L dNTPs was incubated at 65°C for 5 min and 4°C for 2 min. After the addition of 24 *μ*L of 5x first strand buffer, 6 *μ*L of 0.1 mol/L dithiothreitol (DTT), 6 *μ*L of Superscript III (Life Technologies), and 6 *μ*L of RNaseOUT recombinant ribonuclease inhibitor (Life Technologies) into the RNA solution, the mixture was heated at 55°C for 60 min and 70°C for 15 min. Unused primers in the mixture were degraded by Exonuclease I (Affymetrix, Santa Clara, CA). The cDNA was purified with 1.8 volumes of AMPure XP beads (BECKMAN COULTER, Brea, CA).

The polymerase chain reaction (PCR) was performed using the V‐P1 primer set, the T‐PCR‐A primer (5′‐CCATCTCATCCCTGCGTGTC‐3′, with an A‐adaptor at the 5′ end of the sequence), and the Q5 Hot Start High‐Fidelity DNA Polymerase (NEB, Ipswich, MA). The following cycling conditions were used: one cycle of 98°C for 30 sec, 25 cycles of 98°C for 10 sec, 68°C for 10 sec, and 72°C for 30 sec, and one cycle of 72°C for 2 min. For the reverse transcription of RNA from the TILs, 39 *μ*L of a solution containing 3 *μ*g of total RNA from frozen colorectal cancer tissue, 30 pmol of the TCRB‐CA primer, and 0.77 mmol/L dNTPs was heat treated at 65°C for 5 min and 4°C for 2 min. After the addition of 12 *μ*L of 5x first strand buffer, 3 *μ*L of 0.1 mol/L DTT, 3 *μ*L of superscript III, and 3 *μ*L of RNaseOUT recombinant ribonuclease inhibitor into the heat‐treated RNA solution, the mixture was heated at 55°C for 60 min and 70°C for 15 min. Unused primers were degraded and the products were purified as described above. Linear amplification was performed with the purified DNA using the V‐P1 primer set and the Q5 Hot Start High‐Fidelity DNA Polymerase. The following cycling conditions were used: one cycle of 98°C for 30 sec, 10 cycles of 98°C for 10 sec, 68°C for 10 sec, and 72°C for 30 sec, and one cycle of 72°C for 2 min. Linearly amplified products were purified with 1.8 volumes of AMPure XP beads. Subsequent PCR was performed using the T‐PCR‐A primer, the trP1 primer (5′‐CCTCTCTATGGGCAGTCGGTGAT‐3′, with the P1‐adapter sequence at the 5′‐end), and the Q5 Hot Start High‐Fidelity DNA Polymerase. The following cycling conditions were used: one cycle of 98°C for 30 sec, 25 cycles of 98°C for 10 sec, 68°C for 10 sec, and 72°C for 30 sec, and one cycle of 72°C for 2 min. The PCR products were purified with 1.8 volumes of AMPure XP beads.

### DNA sequencing

Sequencing templates were prepared from the same amounts of the DNA libraries from four to six samples with different individual indices using the Ion PI Template OT2 200 Kit v3 or Ion PI Hi‐Q OT2 200 Kit and an Ion OneTouch system (Life Technologies) according to the manufacturer's instructions. The prepared templates were sequenced using an Ion PI Sequencing 200 Kit v3 or Ion PI HI‐Q Sequencing 200 Kit and the Ion Proton sequencer (Life Technologies). Torrent Suite 4.0 to 5.0 software (Life Technologies) was used to convert the raw signals into base calls and to extract the FASTQ files of the sequencing reads.

Human leucocyte antigen (HLA) typing was performed with the Ion Torrent PGM using a protocol developed in our laboratory (manuscript in preparation).

### Data processing

Reads in the FASTQ format were divided using 5‐bp indices for individual assignments. Sequences between the 5‐bp indices and spacer sequences were obtained as barcode tags. Reads with a total length of the spacer and the following sequence shorter than 150 bases were discarded. The monitoring and removal of erroneous barcode tags were performed as described previously 12. After the removal of erroneous tags that had fewer reads than the threshold, the reads from tags with the same barcode were aligned using the multiple alignment program MUSCLE 14, 15, and consensus sequences were created with the majority base (>80%) at each position. If more than 50 reads were obtained, the longest 50 reads were analyzed.

To find the CDR3 sequences in the consensus sequences, we searched the J‐ and V‐regions of TCRB in the consensus sequences using BLAT 16 and the reference sequences in the international ImMunoGeneTics information system (IMGT: http://www.imgt.org/). The definition of CDR3 was based on the IMGT web site (the sequence between the cysteine in the V‐region and the phenylalanine in the J‐region). The nucleotides (nt) of the consensus sequences were converted into amino acids (aa), and aberrant sequences with frame shifts or termination codons were discarded.

## Results

### Outline of the method

**Figure 1:**
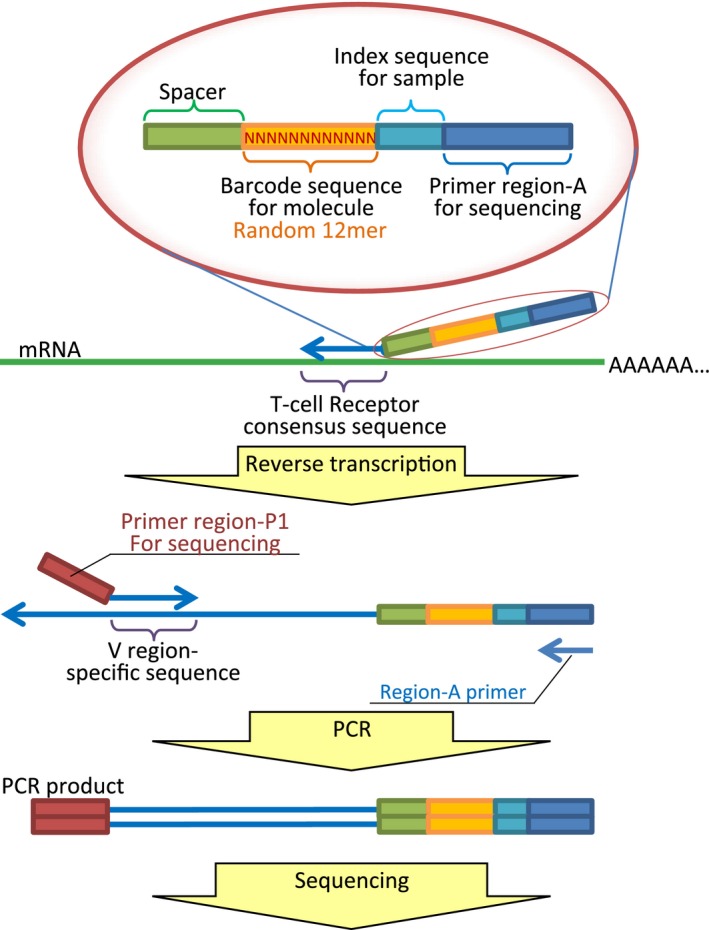
The schematic representation of NOIR‐SS for TCRB. NOIR‐SS, non‐overlapping integrated read sequencing system; TCRB, T‐cell receptor beta chain.

**Figure 2:**
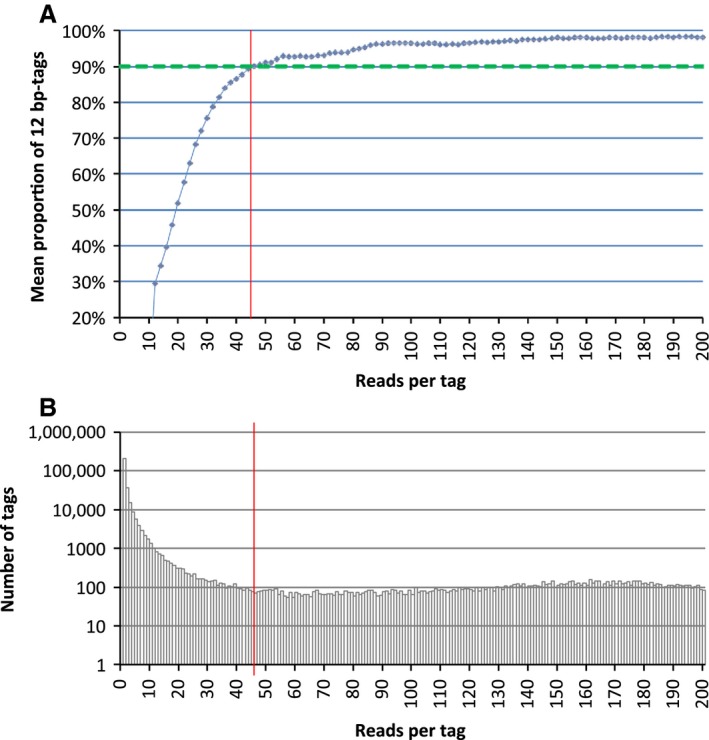
Monitoring the errors in the barcode sequence tags. (A) Distribution of reads per barcode tag. Vertical axis: number of different barcode tags. Horizontal axis: number of reads per tag. (B) The mean proportion of 12‐bp barcode tags after including the 11‐ and 13‐bp tags that differed from a 12‐bp tag only by the insertion or deletion of a single base. Red line, threshold for the removal of reads.

The schematic representation of NOIR‐SS for TCRB is shown in Figure 1. We designed a primer with the barcode sequence (N_12_) for the C region of TCRB. This primer was used for reverse transcription. For the opposite site, a set of PCR primers whose sequences covered the entire V‐region was prepared as previously described 10. PCR amplification was performed after reverse transcription with the primer external to the barcode sequence. The final PCR product was subjected to massively parallel sequencing with an Ion Torrent Proton sequencer. Consensus reads were built using the barcode sequences. The main problem with barcode technology is the errors introduced into the barcode sequences. Because insertion/deletion errors are dominant in the Ion Torrent sequencers, errors in the barcode tags can be monitored by the size of the tags. As revealed in the previous experiment 12, the erroneous barcode tags were accumulated in the fraction of low numbers of reads. Thus, the fraction whose error‐free tags occupied <90% was removed (Fig. 2A). As shown in Figure 2B, the erroneous tags occupied the majority of the tags. This process enabled counting of the sequenced molecules, thereby preventing labeling of individual molecules with multiple barcode tags. This feature is the main advantage of the use of NOIR‐SS over other molecular barcode technologies, including those used for immunorepertoires 17, 18.

### Estimation of the size of the TCRB repertoire in PBLs

We estimated the size of the TCRB repertoire in PBLs and TILs using an abundance‐based coverage estimator (ACE) 19. ACE is regarded as one of the most accurate of the richness estimators (the mathematical methods used to estimate the number of species from limited samplings). ACE estimates the entire TCRB repertoire from the numbers of CDR3 sequences that appear as 1–10 molecules. The ability of NOIR‐SS to count the number of sequenced molecules justified the use of ACE.

Sequencing results and the estimated numbers of the unique CDR3 sequences in PBLs from four healthy individuals (K1, S1, N1, and H1) are shown in Table 1. The estimated numbers of PBLs based on the amount of RNA were 1.6–3.7 × 10^5^.

**Table 1:** Estimation of TCRB repertoires in PBLs and TILs using NOIR‐SS

Lymphocyte	Patient ID	Sequence reads	Molecules (identified with barcode tags)	Number of unique CDR3 nt sequences	Number of unique CDR3 aa sequences	Estimation of TCRB nt repertoire	Estimation of TCRB aa repertoire
PBL	K1	33,068,235	194,398	79,309	76,478	608,664	491,345
PBL	S1	33,520,759	173,962	110,060	104,709	1,003,098	731,011
PBL	H1	18,190,861	80,595	64,227	62,408	955,413	676,582
PBL	N1	20,668,305	88,999	69,253	67,120	731,748	557,577
TIL	CC07	80,405,372	499,732	70,850	68,352	179,297	165,593
TIL	CC13	99,036,747	232,632	51,032	49,671	144,670	135,636
TIL	CC23	63,296,166	459,229	83,294	79,786	223,757	202,616
TIL	CC38	93,672,035	239,520	35,126	34,458	90,228	86,196

TCRB, T‐cell receptor beta chain; PBLs, peripheral blood lymphocytes; TILs, tumor‐infiltrating lymphocytes; NOIR‐SS, non‐overlapping integrated read sequencing system; CDR3, complementarity‐determining region 3.

In previous studies, the most accurate estimation was performed by Warren et al. 13; therefore, this estimation served as the reference. The authors identified 1,061,522 TCRB DNA types in a healthy individual; this figure is similar to the value estimated for S1 and H1.

### Estimation of the size of the TCRB repertoire in TILs

**Figure 3:**
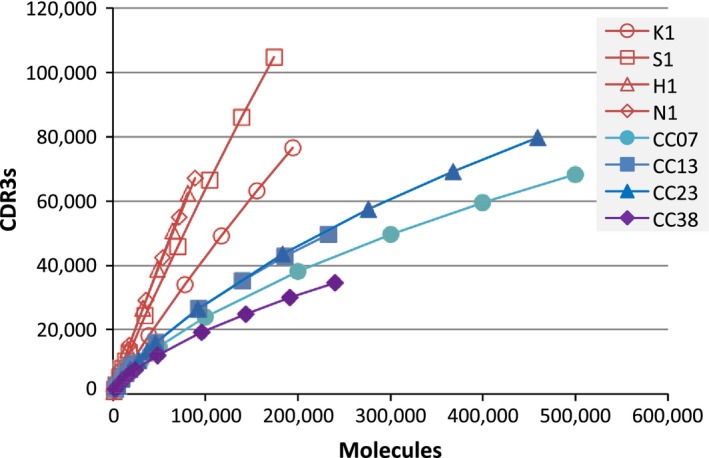
Rarefaction curves. Horizontal axis: number of molecules randomly selected from each pool of sequenced molecules. Vertical axis: number of TCRB CDR3 nucleotide sequences identified. TCRB, T‐cell receptor beta chain; CDR3, complementarity‐determining region 3.

First, we compared the TCRB repertoires of TILs in four colorectal cancer tissues (CC07, CC13, CC23, and CC38) with the TCRB repertoires of PBLs described above using rarefaction curves (Fig. 3). The patient information is shown in Table S2. The repertoires of the TILs were significantly smaller than those of the PBLs (*t*‐test, *P* = 0.0035). Second, we estimated the sizes of the TCRB repertoires in the TILs with ACE. The results are shown in Table 1. The sizes of the TCRBs in the TILs ranged from approximately one‐tenth to one‐third of the sizes in the PBLs.

To demonstrate the advantages of NOIR‐SS, we compared NOIR‐SS to conventional next‐generation sequencing, that is, the original output sequences from the sequencer. We used subpopulations of CC13 sequence data. The estimated size of the TCRB repertoire with NOIR‐SS was consistent and independent of the amount of sequence data (Fig. S1). In contrast, the estimated size of the TCRB repertoire with conventional sequencing was dependent on the amount of sequence data and was not consistent (Fig. S1).

### Comparison of TCRB repertoires in PBLs and TILs

**Figure 4:**
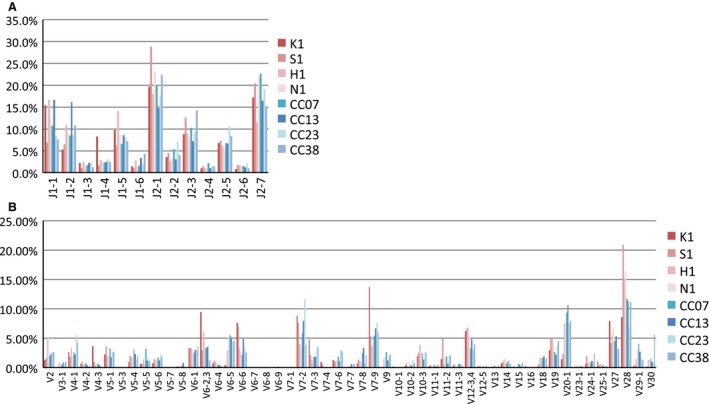
Usage of J‐ and V‐regions in PBLs and TILs. (A) J‐regions. (B) V‐regions. PBLs, peripheral blood lymphocytes; TILs, tumor‐infiltrating lymphocytes.

**Figure 5:**
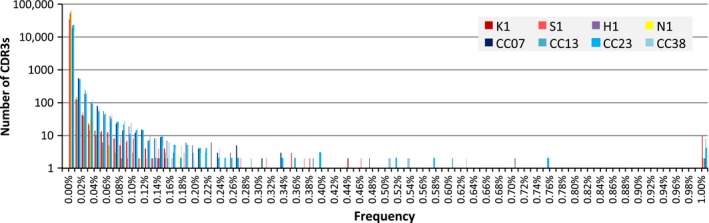
Frequency fractions of CDR3 amino acid sequences. A total of 100,000 molecules were selected from each pool of sequenced molecules, and the numbers of TCRB amino acid types of each frequency fraction were deduced. CDR3, complementarity‐determining region 3; TCRB, T‐cell receptor beta chain.

Next, we compared TCRB repertoires in PBLs and TILs according to several factors. First, we examined the usage of TCRB J‐ and V‐regions. The descriptive statistics of the identified J‐regions and V‐regions are shown in Figure 4. The usage of J‐ and V‐regions was similar between PBLs and TILs. Second, we compared the distributions of CDR3 sequence abundances. The relationship between the numbers of CDR3 aa sequences and their abundance is shown in Figure 5. In the fractions accounting for more than 0.01%, the numbers of TIL CDR3 aa sequences were significantly larger than those of PBLs (Welch *t*‐test, *P* = 0.03). We divided the CDR3 aa sequences into three classes (low, middle, and high) based on their abundances (Table 2). In TILs, the fractions of CDR3 aa sequences belonging to the middle and high abundance classes were larger than those in the PBLs. Both in PBLs and in TILs, fewer nt sequences were shared by the four samples compared to aa sequences (Table 2). This phenomenon could be because a single shared aa sequence corresponded to multiple CDR3 nt sequences.

**Table 2:** The numbers of unique CDR3 nt and aa sequences grouped by the abundance

	K1	S1	H1	N1	Shared by all PBL samples	CC07	CC13	CC23	CC38	Shared by all TIL samples
Nucleotide										
Low <0.01%	78,990 (99.6)	109,723 (99.69)	64,002 (99.65)	68,985 (99.61)	35	69,619 (98.26)	49,927 (97.83)	82,233 (98.73)	33,903 (96.52)	6
Middle 0.01–0.1%	269 (0.34)	282 (0.26)	200 (0.31)	254 (0.37)	0	1106 (1.56)	1028 (2.01)	955 (1.15)	1112 (3.17)	0
High ≥0.1%	50 (0.06)	55 (0.05)	25 (0.04)	14 (0.02)	0	125 (0.18)	77 (0.15)	106 (0.13)	111 (0.32)	0
Amino acids										
Low <0.01%	76,161 (99.59)	104,363 (99.67)	62,173 (99.62)	66,832 (99.57)	57	67,123 (98.2)	48,552 (97.75)	78,727 (98.67)	33,230 (96.44)	182
Middle 0.01–0.1%	265 (0.35)	292 (0.28)	210 (0.34)	274 (0.41)	0	1103 (1.61)	1041 (2.1)	952 (1.19)	1117 (3.24)	0
High ≥0.1%	52 (0.07)	54 (0.05)	25 (0.04)	14 (0.02)	0	126 (0.18)	78 (0.16)	107 (0.13)	111 (0.32)	0

The abundance class of shared CDR3 aa sequences were minimum of the four samples. Figures in parenthesis, fraction of unique CDR3 (%). nt, nucleotide; aa, amino acid; CDR3, complementarity‐determining region 3; PBL, peripheral blood lymphocyte; TIL, tumor‐infiltrating lymphocyte.

### CDR3 aa sequences shared by multiple patients

CDR3 aa sequences shared by multiple patients are of particular interest because they represent responses to common antigens in different patients. Therefore, we analyzed colorectal cancers from an additional 15 patients (see Table S2 for clinical characteristics and Table S3 for HLA type) by low coverage sequencing, with the numbers of identified molecules ranging from 9000 to 52,000. It should be noted that this additional data mainly provided CDR3 aa sequences in the high and middle abundance classes because the number of sequenced molecules were not enough to characterize CDR3 aa sequences belonging to the rare class. The number of shared CDR3 aa sequences decreased as the number of patients increased. There were no TCRB aa types shared by more than 12 patients (Table 3).

**Table 3:** CDR3 aa sequences appearing in multiple samples

	Two patients	Three patients	Four patients	Five patients	Six patients	Seven patients	Eight patients	Nine patients	10 patients	11 patients	12 patients
CDR3 aa sequences appearing in each category in all patients											
Low <0.01%	8659	1715	508	190	80	36	14	5	1	0	1
Middle 0.01–0.1%	218	6	2	0	0	0	0	0	0	0	0
High ≥0.1%	2	0	0	0	0	0	0	0	0	0	0
CDR3 aa sequences appearing in each category at least in one patient											
Low <0.01%	6240	945	187	43	11	2	0	0	0	0	0
Middle 0.01–0.1%	2404	703	299	128	59	27	11	3	1	0	0
High ≥0.1%	235	73	24	19	10	7	3	2	0	0	1

CDR3, complementarity‐determining region 3.

The 5, 1, and 1 CDR3 aa sequences shared by nine, 10, and 12 patients, respectively, revealed some basic characteristics of the shared sequences; the nt and aa composition and V‐ and J‐region information are provided in Table S4. Each CDR3 aa sequence was obtained from a different nt sequence through convergent recombination. Every CDR3 aa sequence used the same J‐region, but usage of the V‐regions was not consistent. No CDR3 aa sequence was consistently associated with any HLA type. There was no apparent association between J‐ and V‐regions and HLA types either. One CDR3 aa sequence was matched to a sequence (accession number, DQ473364.1) 20, found in a patient of celiac syndrome, which was deposited in the NCBI protein database.

## Discussion

Recent advances in high‐throughput sequencing technologies have enabled comprehensive analysis of immunoreceptors. In particular, TCRB is the foremost target of comprehensive sequencing due to its involvement in cellular immunity and its high variability. In this report, we characterized the basic properties of TILs as a molecular population through large‐scale sequencing of TCRB CDR3 regions, and revealed that the estimated size of the TCRB repertoire in TILs was significantly smaller than that in PBLs. The frequencies of the TCRBs belonging to the high and middle abundance classes were elevated in TILs.

The major problem with sequencing immunorepertoires is sequencing errors; the output sequences of massively parallel DNA sequencers introduce bias during the template preparation step, and may not accurately represent the original molecular population. Various computational methods have been used to overcome this problem 21. However, evaluating these methods is difficult. Experimentally, the barcode technology has an advantage and will reduce computational efforts. In terms of experimental strategy, the most accurate estimation of the size of the TCRB repertoire was reported by Warren et al. 13. Instead of the barcode tags used in our study, the authors used J fragments as an indicator of read errors and removed fractions with <96% as erroneous J fragment sequences. They performed exhaustive sequencing (i.e., sequencing TCRB CDR3 regions from two blood samples of a single individual until saturation). Although the use of richness estimators has not necessarily been popular, Qi et al. estimated the size of the TCRB repertoire using a richness estimator 9. Their technical problem was the use of raw sequence data for counting. This bias was removed in our study using the barcode technology implemented in NOIR‐SS. Shugay et al. also applied the barcode technology for sequencing TCRB repertoires 17. They did not consider the errors introduced into barcode sequences, and applied arbitrary cuts for removal of tags with small numbers of reads. Their approach had the same effect for removal of sequencing errors, but lacked the ability of absolute quantitation, which might add important information in the analysis of clinical samples. Both approaches need more sequencing reads than conventional sequencing, but cost would not be a serious problem due to rapid advances in the sequencing technologies.

Sherwood et al. reported the application of high‐throughput sequencing to the TCRB repertoire in colorectal cancer and adjacent mucosal tissues 22. Their size estimation was smaller than ours (100‐fold lower than in the PBLs). However, because their size estimation was the secondary objective and not the primary design and was based on smaller scale conventional sequencing, the finding cannot be adequately compared with our results. The detailed analysis of TILs in ovarian carcinoma and PBLs from the same patients revealed that TCRB repertoires in TILs show strong similarity throughout each tumor, and are distinct from those in PBLs 23. This information complements our results, which suffer from some limitations because we compared repertoires sampled from TILs and PBLs of different individuals.

Individual responses to antigens may be classified into personal or “private” or shared or “public” TCR sequences. Public TCR types are frequent in persistent viral infections 24, 25, 26. In general, the growth of malignant tumors is a long‐term process, and in this respect is similar to persistent virus infections. In mice, a comprehensive analysis of TCRB CDR3 sequences in peripheral blood revealed that any two mice shared 10.5% of their expressed CDR3 aa sequences on average. Additionally, there were a considerable number of “public” CDR3 sequences (defined as those that appeared in most of the recipient mice) that were generally abundant 27. The fraction of shared CDR3 aa sequences was much lower in our study, and no such “public” CDR3 sequences were identified. The difference could be due to genetic factors, including species differences and the identical genetic background of the mice. This mouse analysis also revealed that “public” CDR3 sequences represented a high fraction of those previously associated with autoimmune, allograft, and tumor‐related reactions, but not with antipathogen‐related reactions. In our case, the number of shared CDR3 aa sequences was too small for further speculation.

The main interest in TCRB repertoire sequencing is its clinical application. One interesting example is temporal analysis of the TCRB repertoire during treatment with Cytotoxic T‐lymphocyte associated protein 4 (CTLA‐4) blockers on melanoma 28. The analysis revealed that temporal changes were varied among patients, but identified that maintenance of high‐frequency clones at base lines was associated with longer survival. Recent discovery of correlation between the efficacy of CTLA‐4 blockers and neoantigens appeared by mutations 29 raises the possibility of a link between the high‐frequency clones and the neoantigens.

As previously described 13, 27, 30, shared CDR3 sequences were not necessarily associated with specific HLA types. The CDR1 and CDR2 segments of the TCR, which are expressed on the V‐region segments of the TCR outside of the CDR3 region, interact directly with the MHC molecule 31, 32. In contrast, CDR3 segments are directly associated with antigen epitope recognition. The results of the comprehensive sequence analysis agreed with the previous knowledge.

Bioinformatics should play important roles in the extension of the high‐throughput sequencing strategy to functional analysis. A plausible approach is the identification of CDR3 sequences correlated with phenotypic parameters, including clinical parameters such as prognosis or drug responses. This approach would directly identify prognostic or predictive biomarkers and select CDR3 sequences for further molecular analysis. For this approach, it is important to generate a comprehensive collection of CDR3 sequences shared by multiple patients. The size of such CDR3 sequences in TILs are not likely to be as large as those found in mice, and the screening of a larger number of patients may be necessary to cover the entire population.

## Conflict of Interest

None declared.

## Supporting information


**Figure S1.** Comparison of conventional massively parallel sequencing and NOIR‐SS for the estimation of TCRB nucleotide (nt) repertoire sizes. Horizontal axis: numbers of reads used for estimation. Vertical axis: number of TCRB CDR3 nt sequences identified.

**Table S1.** Sequences of the V‐region primers.

**Table S2.** Colorectal cancer patient information.

**Table S3.** Colorectal cancer patient information (HLA type).

**Table S4.** CDR3 sequences shared by nine to 12 samples.

## References

[1] Fridman, W. H. , F. Pages , C. Sautes‐Fridman , and J. Galon . 2012 The immune contexture in human tumours: impact on clinical outcome. Nat. Rev. Cancer 12:298–306. 22419253 10.1038/nrc3245

[2] deLeeuw, R. J. , S. E. Kost , J. A. Kakal , and B. H. Nelson . 2012 The prognostic value of FoxP3+ tumor‐infiltrating lymphocytes in cancer: a critical review of the literature. Clin. Cancer Res. 18:3022–3029. 22510350 10.1158/1078-0432.CCR-11-3216

[3] Galon, J. , A. Costes , F. Sanchez‐Cabo , A. Kirilovsky , B. Mlecnik , C. Lagorce‐Pages , et al. 2006 Type, density, and location of immune cells within human colorectal tumors predict clinical outcome. Science 313:1960–1964. 17008531 10.1126/science.1129139

[4] Coulie, P. G. , B. J. Van den Eynde , P. van der Bruggen , and T. Boon . 2014 Tumour antigens recognized by T lymphocytes: at the core of cancer immunotherapy. Nat. Rev. Cancer 14:135–146. 24457417 10.1038/nrc3670

[5] Dudley, M. E. , and S. A. Rosenberg . 2003 Adoptive‐cell‐transfer therapy for the treatment of patients with cancer. Nat. Rev. Cancer 3:666–675. 12951585 10.1038/nrc1167 PMC2305722

[6] Pardoll, D. M. 2012 The blockade of immune checkpoints in cancer immunotherapy. Nat. Rev. Cancer 12:252–264. 22437870 10.1038/nrc3239 PMC4856023

[7] Smith‐Garvin, J. E. , G. A. Koretzky , and M. S. Jordan . 2009 T cell activation. Annu. Rev. Immunol. 27:591–619. 19132916 10.1146/annurev.immunol.021908.132706 PMC2740335

[8] Nguyen, P. , J. Ma , D. Pei , C. Obert , C. Cheng , and T. L. Geiger . 2011 Identification of errors introduced during high throughput sequencing of the T cell receptor repertoire. BMC Genomics 12:106. 21310087 10.1186/1471-2164-12-106 PMC3045962

[9] Qi, Q. , Y. Liu , Y. Cheng , J. Glanville , D. Zhang , J. Y. Lee , et al. 2014 Diversity and clonal selection in the human T‐cell repertoire. Proc. Natl. Acad. Sci. USA 111:13139–13144. 25157137 10.1073/pnas.1409155111 PMC4246948

[10] Robins, H. S. , P. V. Campregher , S. K. Srivastava , A. Wacher , C. J. Turtle , O. Kahsai , et al. 2009 Comprehensive assessment of T‐cell receptor beta‐chain diversity in alphabeta T cells. Blood 114:4099–4107. 19706884 10.1182/blood-2009-04-217604 PMC2774550

[11] Zvyagin, I. V. , M. V. Pogorelyy , M. E. Ivanova , E. A. Komech , M. Shugay , D. A. Bolotin , et al. 2014 Distinctive properties of identical twins' TCR repertoires revealed by high‐throughput sequencing. Proc. Natl. Acad. Sci. USA 111:5980–5985. 24711416 10.1073/pnas.1319389111 PMC4000852

[12] Kukita, Y. , R. Matoba , J. Uchida , T. Hamakawa , Y. Doki , F. Imamura , et al. 2015 High‐fidelity target sequencing of individual molecules identified using barcode sequences: de novo detection and absolute quantitation of mutations in plasma cell‐free DNA from cancer patients. DNA Res. 22:269–277. 26126624 10.1093/dnares/dsv010 PMC4535617

[13] Warren, R. L. , J. D. Freeman , T. Zeng , G. Choe , S. Munro , R. Moore , et al. 2011 Exhaustive T‐cell repertoire sequencing of human peripheral blood samples reveals signatures of antigen selection and a directly measured repertoire size of at least 1 million clonotypes. Genome Res. 21:790–797. 21349924 10.1101/gr.115428.110 PMC3083096

[14] Edgar, R. C. 2004 MUSCLE: multiple sequence alignment with high accuracy and high throughput. Nucleic Acids Res. 32:1792–1797. 15034147 10.1093/nar/gkh340 PMC390337

[15] Lloyd, S. , and Q. O. Snell . 2011 Accelerated large‐scale multiple sequence alignment. BMC Bioinformatics 12:466. 22151470 10.1186/1471-2105-12-466 PMC3310909

[16] Kent, W. J. 2002 BLAT–the BLAST‐like alignment tool. Genome Res. 12:656–664. 11932250 10.1101/gr.229202 PMC187518

[17] Shugay, M. , O. V. Britanova , E. M. Merzlyak , M. A. Turchaninova , I. Z. Mamedov , T. R. Tuganbaev , et al. 2014 Towards error‐free profiling of immune repertoires. Nat. Methods 11:653–655. 24793455 10.1038/nmeth.2960

[18] Vollmers, C. , R. V. Sit , J. A. Weinstein , C. L. Dekker , and S. R. Quake . 2013 Genetic measurement of memory B‐cell recall using antibody repertoire sequencing. Proc. Natl. Acad. Sci. USA 110:13463–13468. 23898164 10.1073/pnas.1312146110 PMC3746854

[19] Hughes, J. B. , J. J. Hellmann , T. H. Ricketts , and B. J. Bohannan . 2001 Counting the uncountable: statistical approaches to estimating microbial diversity. Appl. Environ. Microbiol. 67:4399–4406. 11571135 10.1128/AEM.67.10.4399-4406.2001 PMC93182

[20] Meresse, B. , S. A. Curran , C. Ciszewski , G. Orbelyan , M. Setty , G. Bhagat , et al. 2006 Reprogramming of CTLs into natural killer‐like cells in celiac disease. J. Exp. Med. 203:1343–1355. 16682498 10.1084/jem.20060028 PMC2121214

[21] Calis, J. J. , and B. R. Rosenberg . 2014 Characterizing immune repertoires by high throughput sequencing: strategies and applications. Trends Immunol. 35:581–590. 25306219 10.1016/j.it.2014.09.004 PMC4390416

[22] Sherwood, A. M. , R. O. Emerson , D. Scherer , N. Habermann , K. Buck , J. Staffa , et al. 2013 Tumor‐infiltrating lymphocytes in colorectal tumors display a diversity of T cell receptor sequences that differ from the T cells in adjacent mucosal tissue. Cancer Immunol. Immunother. 62:1453–1461. 23771160 10.1007/s00262-013-1446-2 PMC5714653

[23] Emerson, R. O. , A. M. Sherwood , M. J. Rieder , J. Guenthoer , D. W. Williamson , C. S. Carlson , et al. 2013 High‐throughput sequencing of T‐cell receptors reveals a homogeneous repertoire of tumour‐infiltrating lymphocytes in ovarian cancer. J. Pathol. 231:433–440. 24027095 10.1002/path.4260 PMC5012191

[24] Lim, A. , L. Trautmann , M. A. Peyrat , C. Couedel , F. Davodeau , F. Romagne , et al. 2000 Frequent contribution of T cell clonotypes with public TCR features to the chronic response against a dominant EBV‐derived epitope: application to direct detection of their molecular imprint on the human peripheral T cell repertoire. J. Immunol. 165:2001–2011. 10925283 10.4049/jimmunol.165.4.2001

[25] Price, D. A. , J. M. Brenchley , L. E. Ruff , M. R. Betts , B. J. Hill , M. Roederer , et al. 2005 Avidity for antigen shapes clonal dominance in CD8+ T cell populations specific for persistent DNA viruses. J. Exp. Med. 202:1349–1361. 16287711 10.1084/jem.20051357 PMC2212993

[26] Trautmann, L. , M. Rimbert , K. Echasserieau , X. Saulquin , B. Neveu , J. Dechanet , et al. 2005 Selection of T cell clones expressing high‐affinity public TCRs within Human cytomegalovirus‐specific CD8 T cell responses. J. Immunol. 175:6123–6132. 16237109 10.4049/jimmunol.175.9.6123

[27] Madi, A. , E. Shifrut , S. Reich‐Zeliger , H. Gal , K. Best , W. Ndifon , et al. 2014 T‐cell receptor repertoires share a restricted set of public and abundant CDR3 sequences that are associated with self‐related immunity. Genome Res. 24:1603–1612. 25024161 10.1101/gr.170753.113 PMC4199372

[28] Cha, E. , M. Klinger , Y. Hou , C. Cummings , A. Ribas , M. Faham , et al. 2014 Improved survival with T cell clonotype stability after anti‐CTLA‐4 treatment in cancer patients. Sci. Transl. Med. 6:238ra70. 10.1126/scitranslmed.3008211 PMC4558099 24871131

[29] Snyder, A. , V. Makarov , T. Merghoub , J. Yuan , J. M. Zaretsky , A. Desrichard , et al. 2014 Genetic basis for clinical response to CTLA‐4 blockade in melanoma. N. Engl. J. Med. 371:2189–2199. 25409260 10.1056/NEJMoa1406498 PMC4315319

[30] Robins, H. S. , S. K. Srivastava , P. V. Campregher , C. J. Turtle , J. Andriesen , S. R. Riddell , et al. 2010 Overlap and effective size of the human CD8+ T cell receptor repertoire. Sci. Transl. Med. 2:47ra64. 10.1126/scitranslmed.3001442 PMC3212437 20811043

[31] Huseby, E. S. , J. White , F. Crawford , T. Vass , D. Becker , C. Pinilla , et al. 2005 How the T cell repertoire becomes peptide and MHC specific. Cell 122:247–260. 16051149 10.1016/j.cell.2005.05.013

[32] Rudolph, M. G. , R. L. Stanfield , and I. A. Wilson . 2006 How TCRs bind MHCs, peptides, and coreceptors. Annu. Rev. Immunol. 24:419–466. 16551255 10.1146/annurev.immunol.23.021704.115658

