# Supplementary material for: Characterization of the T‐cell receptor beta chain repertoire in tumor‐infiltrating lymphocytes
Source: Cancer Med. 2016 Jul 27;5(9):2513–21. doi: 10.1002/cam4.828 (PMC5055180; doi:10.1002/cam4.828)
Supplement: Supplementary file 1 — Figure S1. Comparison of conventional massively parallel sequencing and NOIR‐SS for the estimation of TCRB nucleotide (nt) repertoire sizes. Horizontal axis: numbers of reads used for estimation. Vertical axis: number of TCRB CDR3 nt sequences identified. [file CAM4-5-2513-s001.pdf]

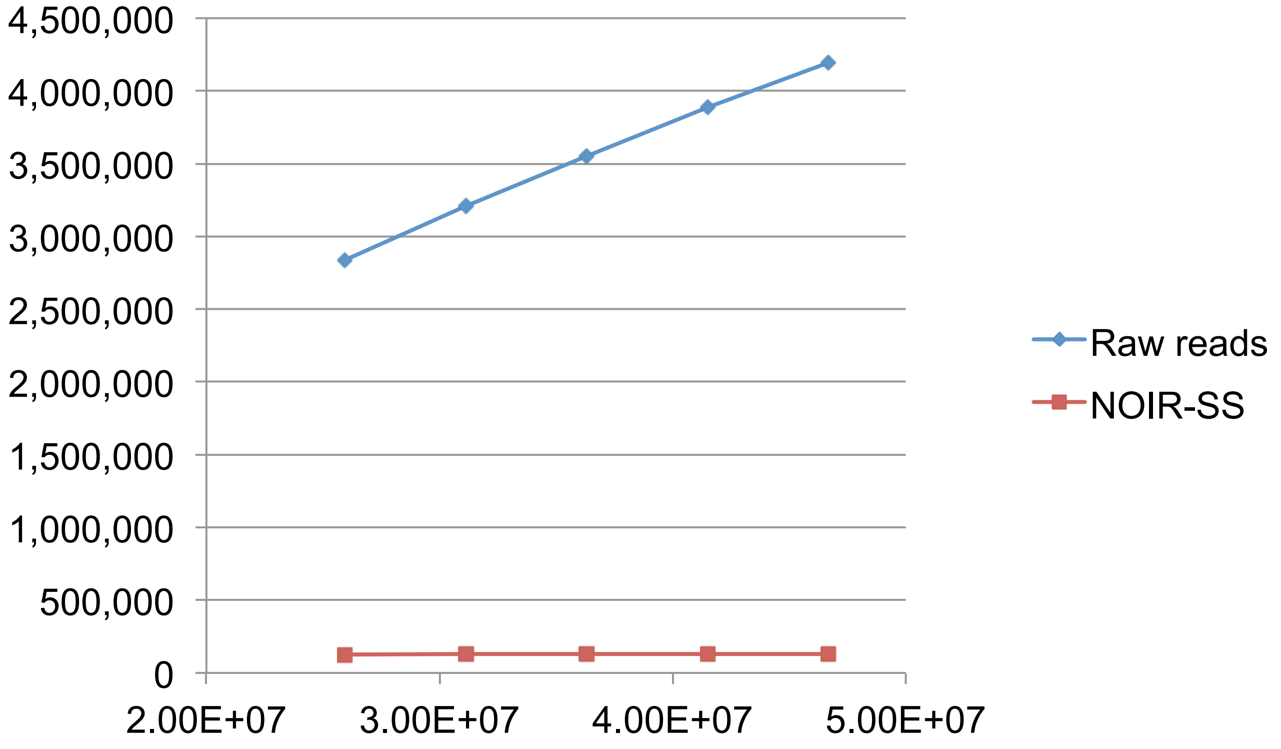

Figure S1. Comparison of conventional massively parallel sequencing and NOIR-SS for the estimation of TCRB nucleotide (nt) repertoire sizes. Horizontal axis, numbers of reads used for estimation. Vertical axis, number of TCRB CDR3 nt sequences identified.
