# Supplementary material for: Characterization of the T‐cell receptor beta chain repertoire in tumor‐infiltrating lymphocytes
Source: Cancer Med. 2016 Jul 27;5(9):2513–21. doi: 10.1002/cam4.828 (PMC5055180; doi:10.1002/cam4.828)
Supplement: Supplementary file 2 — Table S1. Sequences of the V‐region primers. [file CAM4-5-2513-s002.docx]

Table S1. Sequences of the V-region primers.

| oligo_name | oligo_seq |
| --- | --- |
| TRBV3-1_G1 | CCTCTCTATGGGCAGTCGGTGATGCTCACTTAAATCTTCACATCAATTCCCTGG |
| TRBV4-2.3_G1 | CCTCTCTATGGGCAGTCGGTGATCTTATTCCTTCACCTACACACCCTGC |
| TRBV5-1_G1 | CCTCTCTATGGGCAGTCGGTGATGCTCTGAGATGAATGTGAGCACCTTG |
| TRBV6-2.3_G1 | CCTCTCTATGGGCAGTCGGTGATGCTGGGGTTGGAGTCGGCTG |
| TRBV6-4_G1 | CCTCTCTATGGGCAGTCGGTGATCCCTCACGTTGGCGTCTGCTG |
| TRBV6-7_G1 | CCTCTCTATGGGCAGTCGGTGATCCCCTCAAGCTGGAGTCAGCTG |
| TRBV7-2_G1 | CCTCTCTATGGGCAGTCGGTGATCACTCTGACGATCCAGCGCACAC |
| TRBV7-3_G1 | CCTCTCTATGGGCAGTCGGTGATCTCTACTCTGAAGATCCAGCGCACAG |
| TRBV7-7_G1 | CCTCTCTATGGGCAGTCGGTGATCCACTCTGACGATTCAGCGCACAG |
| TRBV9_G1 | CCTCTCTATGGGCAGTCGGTGATGCACTCTGAACTAAACCTGAGCTCTCTG |
| TRBV11-1.3_G1 | CCTCTCTATGGGCAGTCGGTGATCCACTCTCAAGATCCAGCCTGCAG |
| TRBV17_G1 | CCTCTCTATGGGCAGTCGGTGATCTTCCACGCTGAAGATCCATCCCG |
| TRBV19_G1 | CCTCTCTATGGGCAGTCGGTGATCCTCTCACTGTGACATCGGCCC |
| TRBV23-1_G1 | CCTCTCTATGGGCAGTCGGTGATCAGCCTGGCAATCCTGTCCTCAG |
| TRBV30_G1 | CCTCTCTATGGGCAGTCGGTGATCGGCAGTTCATCCTGAGTTCTAAGAAGC |
| TRBV2_G2 | CCTCTCTATGGGCAGTCGGTGATTCAAATTTCACTCTGAAGATCCGGTCCACAA |
| TRBV4-1_G2 | CCTCTCTATGGGCAGTCGGTGATCTTAAACCTTCACCTACACGCCCTGC |
| TRBV5-3_G2 | CCTCTCTATGGGCAGTCGGTGATGCTCTGAGATGAATGTGAGTGCCTTG |
| TRBV6-5_G2 | CCTCTCTATGGGCAGTCGGTGATGCTCAGGCTGCTGTCGGCTG |
| TRBV6-6_G2 | CCTCTCTATGGGCAGTCGGTGATCGCTCAGGCTGGAGTTGGCTG |
| TRBV6-9_G2 | CCTCTCTATGGGCAGTCGGTGATCGCTCAGGCTGGAGTCAGCTG |
| TRBV7-1_G2 | CCTCTCTATGGGCAGTCGGTGATCCACTCTGAAGTTCCAGCGCACAC |
| TRBV7-4_G2 | CCTCTCTATGGGCAGTCGGTGATCCACTCTGAAGATCCAGCGCACAG |
| TRBV10-2_G2 | CCTCTCTATGGGCAGTCGGTGATCCCCCTCACTCTGGAGTCAGCTA |
| TRBV11-2_G2 | CCTCTCTATGGGCAGTCGGTGATCTCCACTCTCAAGATCCAGCCTGCAA |
| TRBV13_G2 | CCTCTCTATGGGCAGTCGGTGATCATTCTGAACTGAACATGAGCTCCTTGG |
| TRBV14_G2 | CCTCTCTATGGGCAGTCGGTGATCTACTCTGAAGGTGCAGCCTGCAG |
| TRBV18_G2 | CCTCTCTATGGGCAGTCGGTGATGCATCCTGAGGATCCAGCAGGTAG |
| TRBV24-1_G2 | CCTCTCTATGGGCAGTCGGTGATCTCCCTGTCCCTAGAGTCTGCCAT |
| TRBV28_G2 | CCTCTCTATGGGCAGTCGGTGATCTCCCTGATTCTGGAGTCCGCCA |
| TRBV5-4.5.6.7.8_G3 | CCTCTCTATGGGCAGTCGGTGATGCTCTGAGCTGAATGTGAACGCCTTG |
| TRBV6-1_G3 | CCTCTCTATGGGCAGTCGGTGATTCGCTCAGGCTGGAGTCGGCTG |
| TRBV6-8_G3 | CCTCTCTATGGGCAGTCGGTGATCACTCAGGCTGGTGTCGGCTG |
| TRBV7-6_G3 | CCTCTCTATGGGCAGTCGGTGATCACTCTGACGATCCAGCGCACAG |
| TRBV7-8_G3 | CCTCTCTATGGGCAGTCGGTGATCCACTCTGAAGATCCAGCGCACAC |
| TRBV7-9_G3 | CCTCTCTATGGGCAGTCGGTGATCACCTTGGAGATCCAGCGCACAG |
| TRBV10-1_G3 | CCTCTCTATGGGCAGTCGGTGATCCCCTCACTCTGGAGTCTGCTG |
| TRBV10-3_G3 | CCTCTCTATGGGCAGTCGGTGATCCTCCTCACTCTGGAGTCCGCTA |
| TRBV12-3.4.5_G3 | CCTCTCTATGGGCAGTCGGTGATCCACTCTGAAGATCCAGCCCTCAG |
| TRBV15_G3 | CCTCTCTATGGGCAGTCGGTGATGATAACTTCCAATCCAGGAGGCCGAACA |
| TRBV16_G3 | CCTCTCTATGGGCAGTCGGTGATCTGTAGCCTTGAGATCCAGGCTACGA |
| TRBV20-1_G3 | CCTCTCTATGGGCAGTCGGTGATCTTGTCCACTCTGACAGTGACCAGTG |
| TRBV25-1_G3 | CCTCTCTATGGGCAGTCGGTGATCCCTGACCCTGGAGTCTGCCA |
| TRBV27_G3 | CCTCTCTATGGGCAGTCGGTGATCCCTGATCCTGGAGTCGCCCA |
| TRBV29-1_G3 | CCTCTCTATGGGCAGTCGGTGATCTAACATTCTCAACTCTGACTGTGAGCAACA |
